# Integrated clinical and genomic characterization of Acinetobacter baumannii causing bloodstream infections in a Chinese tertiary hospital

**DOI:** 10.1099/jmm.0.002177

**Published:** 2026-07-07

**Authors:** Jingyun Ye, Kun Ye, Yuqing Lu, Rui Qiu, Liyan Ye, Jiyong Yang, Lifeng Wang

**Affiliations:** 1 Department of Laboratory Medicine, First Medical Center of Chinese PLA General Hospital, Beijing 100853, PR China; 2 Department of Laboratory Medicine, Civil Aviation General Hospital, Beijing 100123, PR China

**Keywords:** *Acinetobacter baumannii*, bloodstream infection, capsule loci, resistance gene, sequence type

## Abstract

**Introduction.** *Acinetobacter baumannii* is an opportunistic pathogen responsible for severe hospital-acquired infections, including bloodstream infections (BSIs). Although major epidemic clones and resistance mechanisms have been described, integrated analyses combining clinical features, patient outcomes and genomic characteristics of BSI isolates remain limited, particularly in China.

**Gap Statement.** Comprehensive analyses integrating clinical outcomes, high-resolution genotyping, capsule locus diversity and their associations with mortality are still lacking.

**Aim.** This study aimed to characterize the clinical characteristics and outcomes of patients with *A. baumannii* BSIs, as well as the phenotypic and genomic characteristics of the causative isolates. Specifically, we sought to identify high-risk clones and their association with 30-day mortality to improve diagnostic and therapeutic strategies.

**Methodology.** A retrospective cohort study was conducted on 151 cases of *A. baumannii* BSIs in a tertiary hospital in China. Isolates were analysed using whole-genome sequencing to determine sequence types (STs), capsule loci and resistance and virulence gene profiles.

**Results.** The 151 isolates were classified into 11 different STs using the Pasteur MLST scheme, with ST2_Pasteur_ predominating (88.1%). One isolate represented a novel Pasteur ST (ST2831_Pasteur_). Using the Oxford MLST scheme, 22 STs were identified, with ST195_Oxford_, ST208_Oxford_ and ST540_Oxford_ being the most common. Four novel Oxford STs (ST2457_Oxford_, ST2458_Oxford_, ST2459_Oxford_ and ST2460_Oxford_) were identified. Twenty-three capsule locus (KL) types were detected, with KL3 being the most prevalent. Phylogenetic analysis showed clear clustering according to STs and KL types. The ST2_Pasteur_ lineage was characterized by the presence of the *OXA-23* gene and carbapenem resistance, as well as an expanded virulence gene repertoire, suggesting enhanced pathogenic potential. The overall 30-day mortality rate was 35.1%. Patients in the death group had shorter hospital stays, fewer surgical interventions and higher rates of complications, invasive procedures and intensive care unit admission. Certain clone combinations, including ST369_Oxford_-KL9, ST938_Oxford_-KL210 and ST195_Oxford_-KL3, were associated with higher mortality rates.

**Conclusion.** ST369_Oxford_-KL9, ST938_Oxford_-KL210 and ST195_Oxford_-KL3 represent high-risk clones associated with *A. baumannii* BSIs. Early identification of ST and capsule type, alongside clinical risk stratification, may improve diagnosis and guide treatment strategies.

## Data Summary

All genome sequences generated in this study have been deposited in the National Center for Biotechnology Information (NCBI) GenBank database under BioProject accession number PRJNA1286014. Due to the large number of isolates (*n*=151), the individual sequence accession numbers are provided in Table S3

## Introduction

*Acinetobacter baumannii* is an aerobic, non-fermenting, Gram-negative opportunistic pathogen that represents a major global public health concern due to its extensive resistance to multiple antibiotics, including carbapenems [1]. In recent years, it has emerged as a leading cause of hospital-acquired infections, particularly in intensive care units (ICUs) and among immunocompromised patients, where it is associated with pneumonia, skin and soft tissue infections, urinary tract infections and bloodstream infections (BSIs) [2,3]. Globally, the annual incidence of BSIs is estimated at ~150 cases per 100,000 population, with a crude 30-day mortality rate of 17%. In the USA, ~250,000 hospital-acquired BSI cases occur each year, nearly half of which arise in ICUs [4]. *A. baumannii* accounts for ~1.6% of healthcare-associated BSIs in the USA, a proportion similar to that reported in Europe. In China, it is responsible for ~2.8% of such infections [5]. Among these, BSIs represent one of the most severe clinical manifestations, often progressing to systemic inflammatory response syndrome, sepsis and multiple organ dysfunction syndrome (MODS), with reported mortality rates ranging from 30 to 76% [6,8].

The clinical severity of *A. baumannii* infection is primarily driven by the combination of multidrug resistance (MDR) and strong environmental persistence, both of which are closely linked to specific sequence types (STs), resistance determinants and virulence-associated genes [9,10]. Globally, ST1 and ST2 in the Pasteur multilocus sequence typing (MLST) scheme, as well as ST208 and ST369 in the Oxford scheme, represent major epidemic lineages [11,14]. ST1 is widely distributed across Asia and Europe, whereas ST2 is globally distributed [15,16]. In terms of drug resistance mechanisms, *β*-lactamase genes, such as *OXA-23*, *OXA-58*, *OXA-24/40* and *NDM-1*, play important roles [17,18]. In addition, virulence determinants play a critical role in pathogenicity, including *bap* and *csuA/BABCDE*, which are involved in biofilm formation and iron acquisition, as well as genes such as *ompA* and *ptk* that mediate immune evasion [19,20]. Despite these advances, a comprehensive understanding of the integrated clinical and genomic characteristics of * A. baumannii* isolates from BSIs remains limited.

In this study, we systematically analysed the clinical features and outcomes of patients with *A. baumannii* BSIs, together with the epidemiological and genomic characteristics of the causative isolates. These findings aim to inform empirical treatment strategies and support for guiding infection efforts by identifying high-risk clones and associated clinical risk factors.

## Methods

### Bacterial strains and clinical information

A total of 151 non-duplicate *A. baumannii* clinical isolates collected between 2016 and 2019 from the First Medical Center of PLA General Hospital were included in this study. Each isolate originated from a distinct patient and was recovered from blood samples. Initial species identification was performed using matrix-assisted laser desorption/ionization-time of flight MS (bioMérieux SA, France), and species identity was subsequently confirmed by whole-genome sequencing (WGS). Clinical data, including demographic characteristics and outcomes, were retrieved from electronic medical records.

Patients were included if they met all of the following criteria: (1) at least one blood culture positive for *A. baumannii* (for patients with repeated positive cultures, only the first isolate was analysed); (2) clinical signs and symptoms consistent with BSI, such as fever, chills, hypotension, leukocytosis or leucopenia; and (3) availability of complete medical records.

### Antimicrobial susceptibility testing

MICs were determined using the VITEK 2 Compact system (BioMérieux, France) and interpreted according to the Clinical and Laboratory Standards Institute (CLSI) guidelines (M100-S35, 2025). The ten antimicrobial agents tested in this study were selected based on their clinical relevance for treating *A. baumannii* infections and their inclusion in the CLSI guidelines (M100-S35). These agents represent the most frequently used antibiotic classes against *A. baumannii*, including *β*-lactam/*β*-lactamase inhibitor combinations (ampicillin/sulbactam, SAM), cephalosporins (ceftazidime, CAZ; cefepime, FEP; ceftriaxone, CRO), carbapenems (imipenem, IPM), aminoglycosides (gentamicin, GEN; tobramycin, TOB), fluoroquinolones (ciprofloxacin, CIP; levofloxacin, LEV) and sulphonamides (trimethoprim/sulfamethoxazole, SXT). *Escherichia coli* ATCC 25922 and *Pseudomonas aeruginosa* ATCC 27853 were used as quality control strains for antimicrobial susceptibility testing. All bacterial strains were stored at −80 °C for subsequent analyses.

### Whole-genome sequencing

Genomic DNA was extracted from all isolates using the DNeasy**^®^** UltraClean**^®^** Microbial Kit (QIAGEN GmbH, Hilden, Germany). WGS was performed on the Illumina HiSeq X Ten platform (Illumina Inc., San Diego, CA, USA) using paired-end libraries with an average insert size of 350 bp. The sequencing coverage is 100×. Raw sequencing reads were assembled using SOAPdenovo (SOAP v2.21). Assembly quality was assessed using N_50_, N_90_ and scaffold number. The *N*_50_, *N*_90_ and scaffold count were used to identify *de novo* characteristics with QUAST v5.3.0 (Table S1, available in the online Supplementary Material). Scaffolds under 250 bp were excluded from downstream analyses.

### Genomic characteristic analysis

Genome-based taxonomic identification was performed using fastANI (v1.33) with a species-level threshold of 95% average nucleotide identity (ANI). All 151 isolates exhibited ANI values >97% (range 97.3–97.9%) against the *A. baumannii* reference genome ATCC 19606 (GCF_009035845.1), confirming their species as *A. baumannii*. STs were assigned using the *A. baumannii* PubMLST database (https://pubmlst.org/organisms/acinetobacter-baumannii) according to both the Pasteur and Oxford MLST schemes. Novel alleles were submitted to the corresponding MLST databases, and new STs were assigned accordingly. Capsular polysaccharide synthesis loci (K loci, KL) and lipooligosaccharide outer core loci (OCL) were predicted using Kaptive v2.0.1 against the *A. baumannii* database (https://kaptive-web.erc.monash.edu/).

Antimicrobial resistance genes were identified using ResFinder (http://genepi.food.dtu.dk/resfinder). Virulence-associated genes were predicted using VFanalyzer against the *Acinetobacter* genus database in the Virulence Factor Database (VFDB; updated in 2023) (https://www.mgc.ac.cn/cgi-bin/VFs/v5/main.cgi).

All genome sequences generated in this study were deposited in GenBank under BioProject accession number PRJNA1286014.

### Phylogenetic analysis

Single-nucleotide polymorphism-based phylogenetic analysis was conducted using kSNP4.1. The complete genome sequence of *A. baumannii* ATCC 19606 (GCF_009035845.1) was used as the reference genome, as described previously [21]. Phylogenetic trees were visualized using the Interactive Tree of Life (iTOL, https://itol.embl.de/itol.cgi), and annotation tracks were edited using iTOL Editor v1.8.

## Results

### Antimicrobial susceptibility

**Fig. 1.**
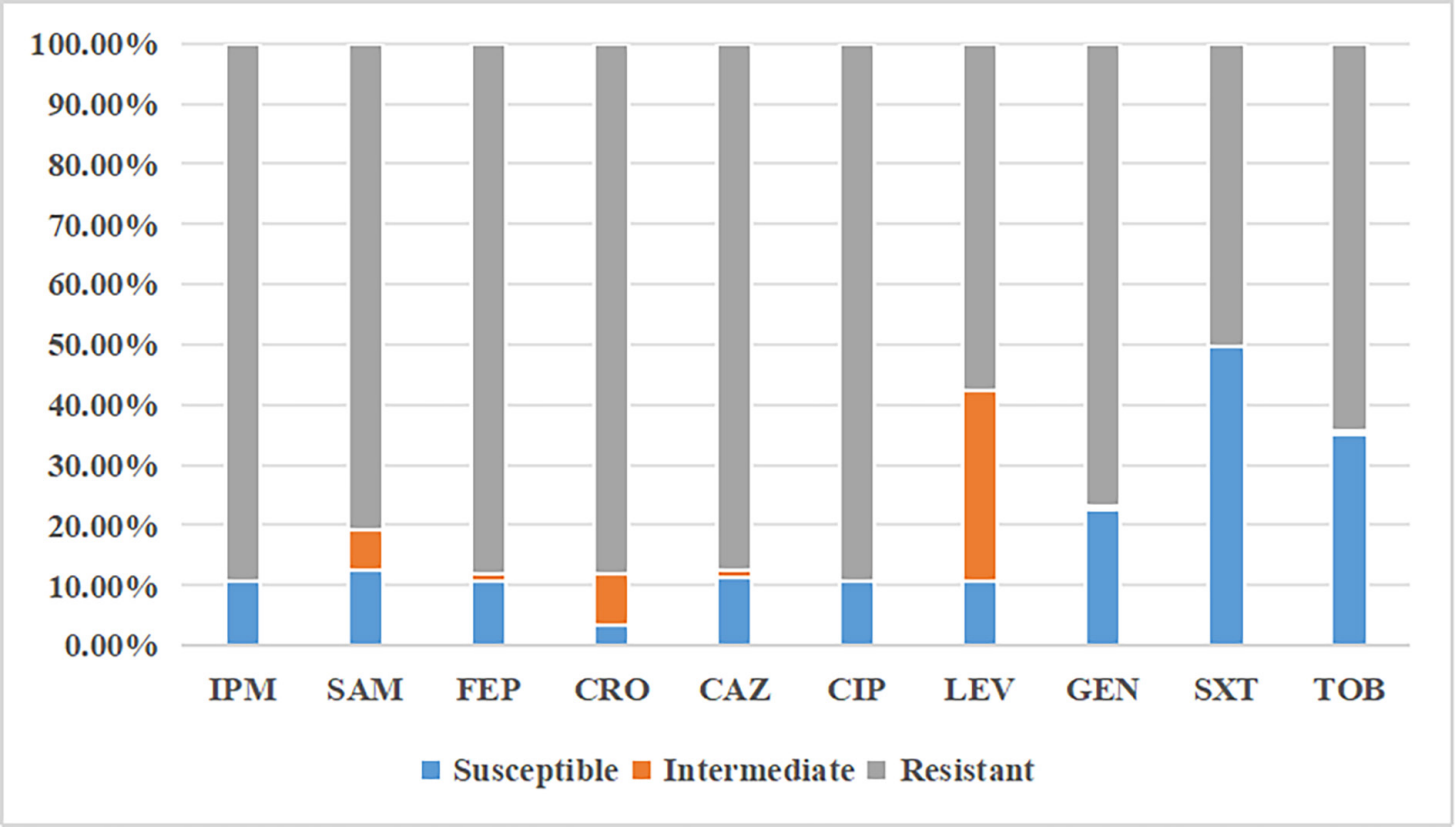
Antibiotic resistance profiles of the 151 *A*. *baumannii* clinical isolates collected between 2016 and 2019. The antibiotics are labelled as follows: imipenem (IPM), ampicillin/sulbactam (SAM), cefepime (FEP), ceftriaxone (CRO), ceftazidime (CAZ), ciprofloxacin (CIP), levofloxacin (LEV), gentamicin (GEN), trimethoprim/sulfamethoxazole (SXT) and tobramycin (TOB).

The antimicrobial susceptibility profiles of the 151 *A*. *baumannii* clinical isolates against 10 antibiotics from 6 antimicrobial classes are shown in Fig. 1. The highest resistance rate was observed for imipenem (89.4%, 135/151) and ciprofloxacin (89.4%, 135/151), followed by cefepime (88.1%, 133/151), ceftriaxone (88.1%, 133/151), ceftazidime (87.4%, 132/151), ampicillin/sulbactam (80.8%, 122/151), gentamicin (76.8%, 116/151), tobramycin (64.2%, 97/151), levofloxacin (57.6%, 87/151) and trimethoprim/sulfamethoxazole (50.3%, 76/151). MDR was defined as non-susceptibility to at least one agent in three or more antimicrobial classes. Based on this definition, 135 isolates (89.4%) were identified as MDR.

### Genomic characteristics

According to the Pasteur MLST scheme, the 151 *A*. *baumannii* strains were assigned to 11 different STs. ST2_Pasteur_, belonging to the global clone 2 (GC2) lineage, was the predominant type, accounting for 88.1% (133/151) of isolates. Other common STs included ST40_Pasteur_ (3.3%, 5/151), ST33_Pasteur_ (2.6%, 4/151) and ST25_Pasteur_ (1.3%, 2/151). The remaining six STs were each represented by a single isolate. One isolate (aba029) was assigned to a novel Pasteur ST, designated ST2831_Pasteur_.

Because the Oxford MLST scheme provides higher discriminatory power, it was also applied. Under this scheme, the 151 isolates were classified into 22 STs. The three most prevalent Oxford STs were ST195_Oxford_ (23.18%, 35/151), ST208_Oxford_ (15.89%, 24/151) and ST540_Oxford_ (13.91%, 21/151). Four isolates were assigned to novel Oxford STs: ST2457_Oxford_ (aba143), ST2458_Oxford_ (aba180), ST2459_Oxford_ (aba030) and ST2460_Oxford_ (aba029). Notably, the *gdhB* gene in ST2460_Oxford_ was assigned a new allele number, 271.

High capsular diversity was observed among the isolates, with 23 capsule locus (KL) types identified. The most prevalent KL type was KL3 (23.2%, 35/151), followed by KL9 (14.6%, 22/151), KL160 (13.9%, 21/151), KL2 (9.9%, 15/151), KL34 (7.3%, 11/151), KL7 (6.0%, 9/151), KL210 (6.0%, 9/151) and KL72 (3.3%, 5/151). The remaining KL types were detected in fewer than three isolates. Three OCL types were identified: OCL1 (88.1%, 133/151), OCL6 (11.3%, 17/151) and OCL8 (0.7%, 1/151). All ST2_Pasteur_ isolates belonged to OCL1. The distribution of KL and OCL types among the *A. baumannii* isolates is shown in Table S2.

### Phylogenetic analysis

**Fig. 2.**
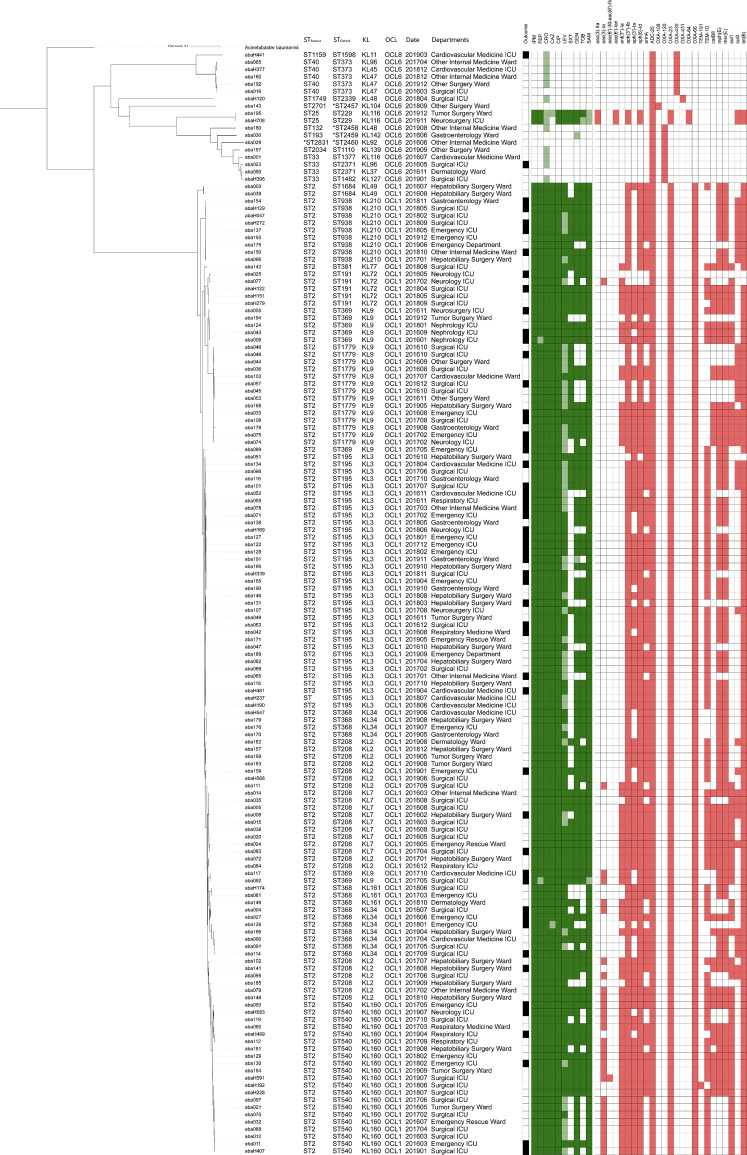
Maximum-likelihood (ML) core-genome phylogenetic tree of the 151 *A*. *baumannii* clinical isolates. Each isolate is annotated with its corresponding Pasteur ST, Oxford ST, KL type, OCL type, isolation date, isolation department, clinical outcome, antibiotic resistance phenotypes and antibiotic resistance (AMR) genes. For clinical outcome, a black square represents death, and a colourless square represents survival. The antibiotics are labelled as follows: imipenem (IPM), cefepime (FEP), ceftriaxone (CRO), ceftazidime (CAZ), ciprofloxacin (CIP), levofloxacin (LEV), trimethoprim/sulfamethoxazole (SXT), gentamicin (GEN), tobramycin (TOB) and ampicillin/sulbactam (SAM). The assigned antibiotic resistance phenotypes are colour coded as dark green for resistance, light green for intermediate resistance and colourless for susceptible. An orange square represents the presence of AMR genes, and a colourless square represents their absence.

**Fig. 3.**
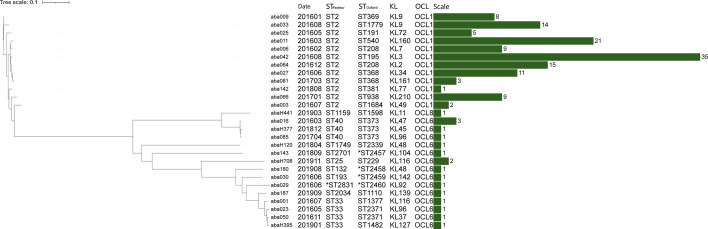
Maximum Likelihood (ML) core-genome phylogenetic tree of the 27 *A*. *baumannii* clinical isolates. Each isolate is annotated with its corresponding Pasteur ST, Oxford ST, KL type, OCL type, isolation date and scale. The scale indicates the number of isolates per Oxford ST and KL type.

A WGS-based phylogenetic tree is shown in Fig. 2. The analysis revealed that the isolates clustered primarily according to STs and KL types, with the population divided into two major lineages: ST2_Pasteur_ and non-ST2_Pasteur_. The ST2_Pasteur_ lineage was subdivided into 12 distinct clusters based on Oxford STs and KL types. The most prevalent clonal combinations were ST195_Oxford_-KL3 (23.18%, 35/151) and ST540_Oxford_-KL160 (13.91%, 21/151). Exceptions were observed within certain Oxford STs: ST208_Oxford_ isolates within the ST2_Pasteur_ lineage were associated with either KL2 (*n*=15) or KL7 (*n*=9), while ST368_Oxford_ isolates were associated with either KL34 (*n*=11) or KL161 (*n*=3) (Fig. 3).

### Genetic determinants of resistance

A broad range of antimicrobial resistance genes associated with multiple drug classes was identified among the *A. baumannii* clinical isolates (Fig. 2). The class C *β*-lactamase gene (*ADC-25*) was identified in all isolates. Class D *β*-lactamases were widely distributed, with seven class D *β*-lactamase genes identified. Intrinsic *OXA-51*-like genes were present in all isolates. The carbapenemase gene *OXA-23* was identified in all 135 carbapenem-resistant isolates, including 133 ST2_Pasteur_ isolates and 2 ST25_Pasteur_ isolates. The *OXA-66* gene was identified in all 133 ST2_Pasteur_ isolates. Additional class D *β*-lactamase genes detected included *OXA-120*, *OXA-430*, *OXA-64*, *OXA-106* and *OXA-431*.

### Virulence factors

**Fig. 4.**
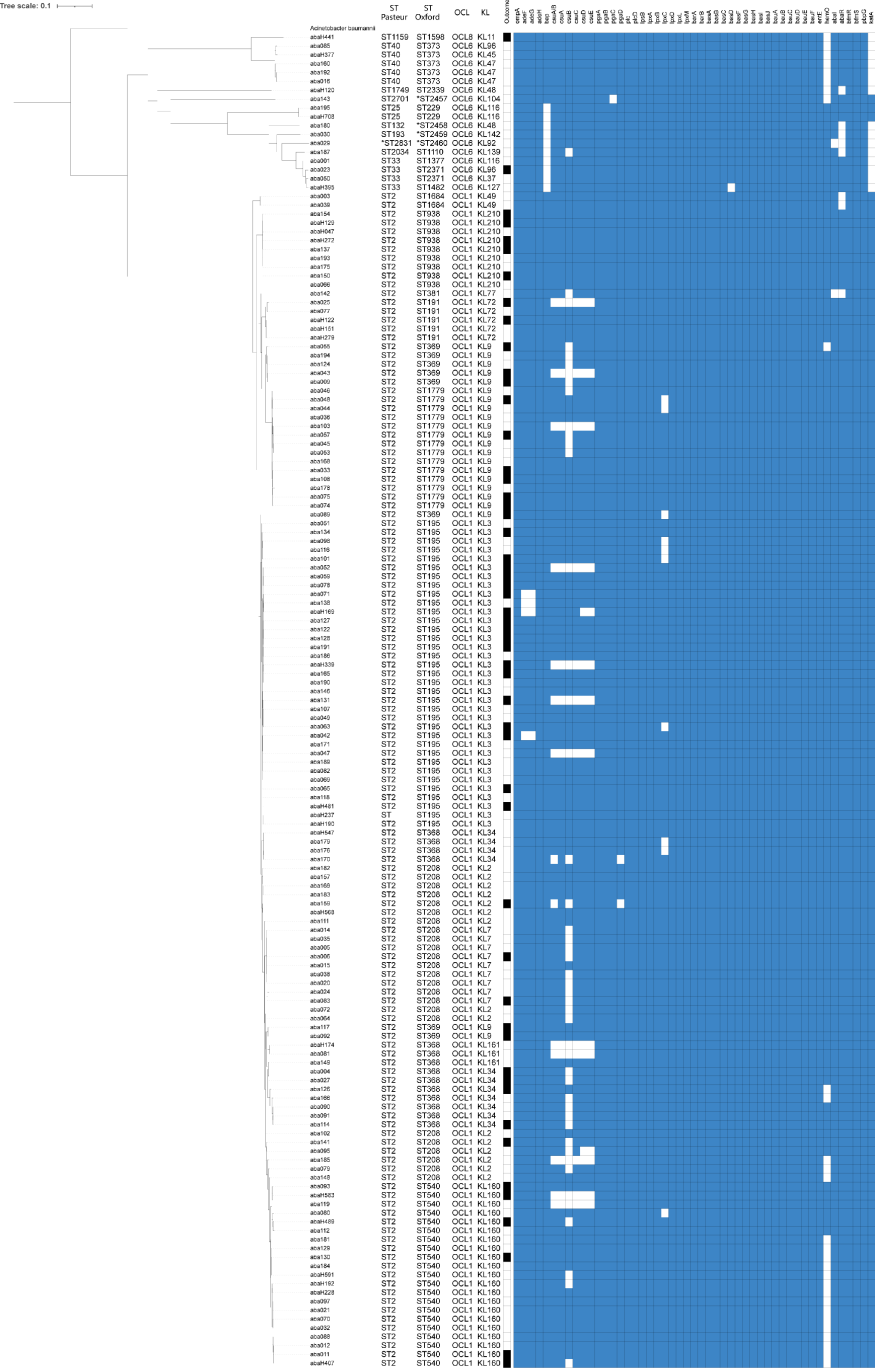
Maximum Likelihood (ML) core-genome phylogenetic tree of the 151 *A*. *baumannii* clinical isolates. Each isolate is annotated with its corresponding Pasteur ST, Oxford ST, KL type, OCL type, clinical outcome and virulence factors. For clinical outcome, a black square represents death, and a colourless square represents survival. Regarding virulence factors, a blue square indicates the presence of virulence factors, and a colourless square indicates their absence.

All 151 *A*. *baumannii* isolates were screened for virulence-associated genes using the VFDB, and the overall distribution profile is shown in Fig. 4. A total of 49 distinct virulence genes were identified across the isolate collection, with each isolate harbouring between 42 and 49 genes. A core set of 32 virulence genes was universally present and involved in multiple pathogenic processes, including adhesion (*ompA*), biofilm formation (*adeH*, *pgaA* and *pgaB*), phospholipase activity (*plc* and *plcD*), immune evasion (*lpsB*, *lpxA*, *lpxB*, *lpxD*, *lpxL* and *lpxM*), iron uptake (*barA*, *barB*, *basA*, *basB*, *basC*, *basF*, *basG*, *basH*, *basI*, *basJ*, *bauA*, *bauB*, *bauC*, *bauD*, *bauE*, *bauF* and *entE*), regulation (*bfmR* and *bfmS*) and serum resistance (*pbpG*). The total number of virulence genes varied across isolates: 57 isolates carried all 49 genes, 52 carried 48 genes and 19 carried 47 genes. Variable gene distribution was observed for several key factors. The *bap* gene was absent in 10 of the 18 non-ST2_Pasteur_ isolates. The *csuA/BABCDE* operon was absent in 12 ST2_Pasteur_ isolates, and the *hemO* gene was not detected in 29 isolates. In contrast, the *katA* gene was conserved in all ST2_Pasteur_ isolates.

### Clinical analysis according to the 30-day outcomes

A total of 151 patients with *A. baumannii* BSI were included in this study. The cohort comprised 111 males and 40 females, with an average age of 56.8 years (range, 2–91 years). Most patients were admitted to the surgical ICU (29.8%, 45/151), followed by the hepatobiliary surgery ward (13.9%, 21/151) and emergency ICU (12.6%, 19/151). Overall, 53 patients died within 30 days after the onset of BSI, yielding a 30-day mortality rate of 35.1%. Comparisons between the survival and death groups are summarized in Table 1. In both groups, most patients were older adults and male. Patients in the survival group had significantly longer hospital stays and were more likely to have undergone surgical treatment than those in the death group (*P*=0.006 and *P*=0.007, respectively). In contrast, ICU admission rate was significantly more frequent in the death group (*P*<0.001). Patients in the death group also had significantly higher rates of complications, including pulmonary infection, septic shock, respiratory failure and MODS (*P*<0.001). Invasive procedures, such as tracheal intubation, intravenous catheterization and gastric tube placement, were more common in the death group than in the survival group (*P*<0.001, *P*=0.007 and *P*=0.005, respectively). By contrast, drainage tube placement was more frequent in the survival group (*P*=0.017). Regarding microbiological findings from other specimens, *A. baumannii* was more frequently isolated from sputum samples in the death group than in the survival group (*P*<0.001), whereas isolation from drainage fluid samples was more common in the survival group (*P*=0.011). Analyses of Oxford ST and KL combinations showed that ST369_Oxford_-KL9, ST938_Oxford_-KL210 and ST195_Oxford_-KL3 were associated with high mortality rates of 75%, 55.5% and 51.4%, respectively. Moreover, the proportions of ST195_Oxford_-KL3 and ST369_Oxford_-KL9 were significantly higher in the death group than in the survival group (*P*=0.021 and *P*=0.023, respectively).

**Table 1.** Clinical characteristics of patients with *A. baumannii* BSI by clinical outcome

Variable	Survival group (*n*=98)	Death group (*n*=53)	*P* value
Male sex, *n* (%)	69 (70.4%)	42 (79.2%)	0.240
Age, mean±sd	55.59±19.39	59.04±18.91	0.292
Comorbidities, *n* (%)			
Pulmonary infection	21 (21.4%)	32 (60.4%)	**<0.001**
Infectious shock	6 (6.1%)	20 (37.7%)	**<0.001**
Respiratory failure	6 (6.1%)	18 (34.0%)	**<0.001**
Multiple organ failure	3 (3.1%)	11 (20.8%)	**<0.001**
Hypertension	32 (32.7%)	27 (50.9%)	**0.028**
Diabetes	15 (15.3%)	13 (24.5%)	0.164
Trauma	16 (16.3%)	6 (11.3%)	0.405
Cancer	41 (41.8%)	14 (26.4%)	0.060
Cardiovascular diseases	25 (25.5%)	22 (41.5%)	**0.043**
Liver function injury	25 (25.5%)	22 (41.5%)	**0.043**
Renal dysfunction	17 (17.3%)	18 (34.0%)	**0.021**
Abdominal inflammation	33 (33.7%)	12 (22.6%)	0.157
Hospitalization time, mean±sd	40.60±32.48	26.94±19.09	**0.006**
Time from check-in to blood culture positive, mean±sd	16.55±21.96	18.08±16.66	0.657
Admission to ICU, *n* (%)	61 (62.2%)	47 (88.7%)	**<0.001**
Surgery, *n* (%)	68 (69.4%)	25 (47.2%)	**0.007**
Invasive operations, *n* (%)			
Fiberoptic bronchoscopy examination	12 (12.2%)	8 (15.1%)	0.622
Tracheal intubation	38 (38.8%)	37 (69.8%)	**<0.001**
Intravenous catheterization	50 (51.0%)	39 (73.6%)	**0.007**
Urinary catheter	34 (34.7%)	20 (37.7%)	0.710
Gastric tube	21 (21.4%)	23 (43.4%)	**0.005**
Drainage tube	55 (56.1%)	19 (35.8%)	**0.017**
Separate *A. baumannii* from other specimens, *n* (%)			
Sputum	35 (36.7%)	37 (69.8%)	**<0.001**
Drainage fluid	29 (29.6%)	6 (11.3%)	**0.011**
Venous catheter	19 (19.4%)	8 (15.1%)	0.511
Other specimens	20 (20.4%)	10 (18.9%)	0.821
Multiple isolation of *A. baumannii* in blood culture, *n* (%)	37 (37.8%)	18 (34.0%)	0.644
Simultaneous isolation of other pathogenic bacteria from blood culture, *n* (%)	21 (21.4%)	12 (22.6%)	0.863
Oxford ST-KL, *n* (%)			
ST195-KL3	17 (17.3%)	18 (34.0%)	**0.021**
ST540-KL160	15 (15.3%)	6 (11.3%)	0.499
ST208-KL2	13 (13.3%)	2 (3.8%)	0.063
ST208-KL7	7 (7.2%)	2 (3.8%)	0.396
ST1779-KL9	8 (8.2%)	6 (11.3%)	0.523
ST938-KL210	4 (4.1%)	5 (9.4%)	0.185
ST368-KL34	7 (7.1%)	4 (7.5%)	0.927
ST369-KL9	2 (2.1%)	6 (11.3%)	**0.023**

Bold values: *P*< 0.05 (statistically significant).

## Discussion

Given the escalating threat posed by *A. baumannii* BSIs and the urgent need for effective management strategies, a comprehensive understanding of the clinical and genomic characteristics of *A. baumannii* isolates is essential [1]. As a major nosocomial pathogen, *A. baumannii* frequently causes severe infections, particularly in immunocompromised patients, and its MDR further complicates treatment [22]. In this study, we analysed 151 patients with *A. baumannii* BSIs and found a 30-day mortality rate of 35.1%, highlighting the substantial clinical burden of these infections [6,8]. Integrated genomic analysis identified ST369_Oxford_-KL9, ST938_Oxford_-KL210 and ST195_Oxford_-KL3 as high-risk clones associated with adverse outcomes.

The predominance of older male patients in both outcome groups is consistent with the recognized susceptibility of vulnerable hospitalized populations to *A. baumannii* infection [23]. The shorter hospital stays observed in the death group, despite higher ICU admission rates, may reflect more rapid disease progression and greater illness severity. This interpretation is supported by higher rates of pulmonary infection, septic shock, respiratory failure and MODS in patients who died [24]. Invasive procedures, including tracheal intubation, intravenous catheterization and gastric tube placement, were also more common in the death group, highlighting their potential role as risk factors by facilitating pathogen colonization and bloodstream invasion [25,26]. Conversely, the higher frequency of drainage tube placement in the survival group may suggest a potential benefit in controlling localized infections and preventing systemic dissemination.

We characterized all *A. baumannii* isolates using the Pasteur and Oxford MLST schemes. Using the Pasteur scheme, we classified the 151 isolates into 11 STs, with ST2_Pasteur_, a member of the globally prevalent GC2 lineage, accounting for 88.1% of the isolates [27,30]. This predominance reflects the exceptional adaptability and persistence of this lineage in hospital environments [31]. The less common types included ST40_Pasteur_, ST33_Pasteur_, ST25_Pasteur_ and the novel ST2831_Pasteur_. Consistent with previous reports, the Oxford scheme exhibited higher discriminatory power, enabling further subdivision of the dominant ST2_Pasteur_ lineage [14]. ST195_Oxford_, ST208_Oxford_ and ST540_Oxford_ were the most prevalent subtypes. Additionally, four novel Oxford STs were identified, including ST2460_Oxford_, which carried a newly assigned *gdhB* allele (271). These findings highlight the ongoing genomic diversification of *A. baumannii* and emphasize the importance of continuous molecular surveillance.

Phylogenetic analysis based on WGS revealed that *A. baumannii* isolates clustered primarily by STs and KL types, confirming these markers as key determinants of population structure [27]. The ST2_Pasteur_ lineage was dominant, accounting for the majority of isolates. As expected, all ST2_Pasteur_ isolates carried the *OXA-23* gene and exhibited carbapenem resistance [32]. Notably, the *OXA-66* gene was universally present in this lineage, suggesting its potential utility as a genetic marker for tracking GC2 [33,34]. The universal presence of *ADC-25* and *OXA-51*-like genes across all isolates confirms their role in intrinsic resistance [33]. Together, these findings indicate that the highly drug-resistant GC2 lineage is a major driver of BSI transmission in our hospital, underscoring the need for targeted infection control.

*A. baumannii* isolates harboured a diverse array of virulence factors involved in adhesion, biofilm formation, immune evasion, iron uptake and stress adaptation. The *ompA* gene, which encodes a major outer membrane adhesin, was detected in all isolates [35,37]. Notably, the *bap* gene, a key regulator of biofilm formation and persistence, was present in all ST2_Pasteur_ isolates but absent in 10 of the 18 non-ST2_Pasteur_ isolates, suggesting a potential role in the persistence and transmission of the dominant lineage [20,38, 39]. In addition, the *katA* gene, which contributes to oxidative stress defence, was detected in all carbapenem-resistant isolates and in one carbapenem-susceptible isolate, indicating a possible association with resistance and its potential utility as a marker for resistant strains [40]. These findings highlight that the convergence of specific virulence determinants (e.g. *bap*) and MDR within the GC2 lineage likely enhances its clinical fitness and pathogenicity.

We acknowledge several limitations of this study. First, the single-centre retrospective design may have introduced selection bias and limited the generalizability of our findings to other institutions or geographic regions. Second, as an observational study, our analysis can identify associations but cannot establish causal relationships between specific bacterial genotypes and patient mortality. Third, the limited number of death events (*n*=53) and small subgroup sizes (e.g. rare STs) preclude reliable multivariate regression analysis and formal adjustment for confounders; we also did not assess statistical power or adjust for multiple comparisons. Therefore, findings for low-frequency clones should be interpreted cautiously. Future prospective multicentre studies incorporating mechanistic investigations are necessary to validate these findings and further elucidate the underlying pathogenic mechanisms. Nevertheless, this study provides important genomic and clinical data that can inform future hypothesis-driven research.

In conclusion, this study reveals the high mortality and extensive drug resistance of *A. baumannii* BSIs, driven largely by the virulent and multidrug-resistant ST2_Pasteur_ lineage. Clinical risk factors such as invasive procedures, ICU admission and septic shock were strongly associated with poor outcomes, underscoring the importance of early recognition and timely intervention. In particular, ST369_Oxford_-KL9, ST938_Oxford_-KL210 and ST195_Oxford_-KL3 were identified as high-risk clonal combinations associated with increased mortality. These findings emphasize the value of integrated clinical and genomic surveillance to inform infection control measures, optimize antimicrobial stewardship and support the development of novel therapeutic strategies against this growing threat.

## Supplementary material

10.1099/jmm.0.002177 Fig. S1.

## Abbreviations

ANI: average nucleotide identity
BSI: bloodstream infection
CLSI: Clinical and Laboratory Standards Institute
GC2: global clone 2
ICU: intensive care unit
iTOL: Interactive Tree of Life
MDR: multidrug resistance
ML: maximum likelihood
MLST: multilocus sequence typing
MODS: multiple organ dysfunction syndrome
NCBI: National Center for Biotechnology Information
OCL: outer core loci
ST: sequence type
VFDB: Virulence Factor Database
WGS: whole-genome sequencing
